# The Association between Risk Perception and Hesitancy toward the Booster Dose of COVID-19 Vaccine among People Aged 60 Years and Older in China

**DOI:** 10.3390/vaccines10071112

**Published:** 2022-07-12

**Authors:** Chenyuan Qin, Wenxin Yan, Liyuan Tao, Min Liu, Jue Liu

**Affiliations:** 1 Department of Epidemiology and Biostatistics, School of Public Health, Peking University, No. 38, Xueyuan Road, Haidian District, Beijing 100191, China; qincy@bjmu.edu.cn (C.Q.); yanwx@bjmu.edu.cn (W.Y.); liumin@bjmu.edu.cn (M.L.); 2 Research Center of Clinical Epidemiology, Peking University Third Hospital, No. 49 Huayuan North Road, Haidian District, Beijing 100083, China; tendytly@163.com; 3 Institute for Global Health and Development, Peking University, Beijing 100871, China; 4 National Health Commission Key Laboratory of Reproductive Health, Peking University, Beijing 100191, China

**Keywords:** COVID-19, vaccination, booster dose, hesitancy, old people

## Abstract

Background: Given the prevalence of the omicron variant and decreased immunity provided by vaccines, it is imperative to enhance resistance to COVID-19 in the old population. We planned to explore the hesitancy rate toward the booster dose of the COVID-19 vaccine and the association between risk perception and the abovementioned rate among people aged 60 and older. Methods: This national cross-sectional study was conducted in mainland China from 25 May to 8 June 2022, targeting people who were 60 years old or above. Four dimensions were extracted from the Health Belief Model (HBM) to assess participants’ perceived risk levels, including perceived susceptibility, perceived severity, perceived barriers, and perceived benefit. An independent Chi-square test was used to compare the vaccine hesitancy rates among different groups stratified by characteristics. Univariable and multivariable logistic regression models were performed to explore the associations between risk perception and hesitancy rate. Results: Of 3321 participants, 17.2% (95% CI: 15.9–18.5%) were hesitant about booster shots of COVID-19 vaccines. Believing that they were ineligible for vaccination due to certain illnesses (38.3%), concern about vaccine safety (32.0%), believing the booster shots were unnecessary (33.1%), and their limitation on movements (28.0%) were the main reasons for vaccine hesitation. Adjusted by all the selected covariates, people with low perception level of susceptibility (aOR = 1.39, 95% CI: 1.00–1.92) and benefit (low: aOR = 3.31, 95% CI: 2.01–5.45; moderate: aOR = 2.23, 95% CI: 1.75–2.85) were less likely to receiving the booster dose, and the same results were found in people with higher perceived barriers (moderate: aOR = 2.67, 95% CI: 2.13–3.35; high: aOR = 2.04, 95% CI: 1.14–3.67). Our estimates were stable in all four models. Conclusions: In total, 17.2% of the people aged 60 years and older in China were hesitant about booster dose of COVID-19 vaccines, and it was closely associated with a lower level of perceived susceptibility and benefit, as well as a higher level of perceived barriers. Concerns about contraindications, vaccine safety, and limited movements were the main reasons for vaccine hesitancy. Targeted public health measure is a priority to improve the understanding of the elderly on their own susceptibility and vulnerability and clear the obstacles to vaccination.

## 1. Introduction

As one of the worst plagues in nearly a century, coronavirus disease 2019 (COVID-19) has caused an incalculable disease and economic burden across the globe [1]. Unfortunately, more than 540.5 million people have been directly affected by this pandemic as of 22 June 2022, with 6.3 million death cases [2]. All viruses, including severe acute respiratory syndrome coronavirus 2 (SARS-CoV-2), the virus that caused COVID-19, changed over time, and five variants of concern (VOCs) have entered the public domain so far [3]. Undoubtedly, the Omicron variant (B.1.1.529) is the currently circulating one, carrying multiple spike mutations with high transmissibility and immune escape [3,4]. Vaccination was thought to be the most cost-effective public health intervention to control infectious diseases and protect people’s lives and property [5]. The old population is usually accompanied by poor immunity and more underlying diseases, making them a high-risk group for SARS-CoV-2 infection and poor prognosis [6,7]. Studies have shown that vaccination against COVID-19 in older adults is effective in reducing severe illness and death, and the safety of the vaccine has also been fully recognized [4,7,8]. Data from the US, Germany, and South Korea all confirmed that prioritizing the oldest people with COVID-19 vaccination could save the most lives and, surprisingly, maximize the remaining life expectancy [9]. 

Vaccine hesitancy, one of the ten major threats to global health in 2019, refers to the reluctance or refusal of people to be vaccinated when available, especially among the older population [10]. According to a report on the fifth wave of COVID-19 in Hong Kong, China, people aged 60 or above accounted for 96% of all deaths, and 88% of the deaths were either unvaccinated or received only one dose of COVID-19 vaccine [11]. Among the old population, the mortality rate was 0.70% for those who were unvaccinated, albeit to 0.02% for people who have completed two or three doses [11]. Obviously, the emergence of VOCs and decreased immunity provided by vaccines could lead to an increase in breakthrough infections, which indicates that receiving a booster dose of the COVID-19 vaccine is imperative [12,13,14]. However, only 26.4% of the world’s population have received the booster doses against COVID-19 [15] till 20 June 2022, and the Joint Prevention and Control Mechanism of the State Council claimed that, as of 16 June 2022, 35.2% of the elderly (≥60 years old) in China still had not received the booster shots [16]. Another critical issue that needs to be highlighted is that the overall level of effective antibodies produced by older people after vaccination was lower than that produced by younger adults [7,17]. 

The health belief model (HBM) is widely used to assess public attitudes toward vaccines and predict their behavior toward vaccination, revealing significant perception factors such as perceived susceptibility, perceived severity, perceived barriers, and perceived benefit [18,19,20]. Risk perception is people’s subjective judgments of a particular risk, which will eventually affect people’s behavior [21,22,23]. Studies have shown that higher levels of knowledge among older people about epidemiological characteristics, the number of deaths, and effective prevention measures of infectious diseases have been shown to be associated with positive behavior change [20,23,24,25]. Perceptions or beliefs about an outbreak are important in deciding to take specific preventive actions [26,27].

Up to now, no study has been performed to specifically investigate the hesitancy toward the booster dose of the COVID-19 vaccine and the association between risk perceptions among the Chinese elderly (≥60 years old). Accelerating global coverage of vaccination in the old population, especially the booster dose, is indeed a priority for achieving herd immunity against COVID-19 [28]. To better understand the elderly’s hesitancy toward the booster dose and provide a theoretical basis for policy making, it is fundamental to conduct an anonymous survey to evaluate the hesitancy rate and the association between risk perception and the abovementioned rate in this specific population.

## 2. Methods

### 2.1. Study Design and Participants

This national cross-sectional survey was conducted based on an online platform called Wen Juan Xing (Changsha Ranxing Information Technology Co., Ltd., Changsha, China). Owing clear personal information (e.g., age, gender, and residence) of nearly 3 million registered members in China, it could accurately deliver questionnaires to the representative respondents we expect. The target participants of this study were senior citizens aged 60 or above in China. For users who have trouble answering questions using electronic devices (without cognitive impairment), they can enlist the help of those around them, and questions could be relayed to the old people by those young adults living with them. Each question was followed by a reminder that the purpose of the questionnaire was to find out the true thoughts and situations of people aged 60 and above.

According to previous studies that examined the willingness of Chinese adults to receive booster shots, 10.56% (95% CI: 6.99–14.14%) were unwilling to receive booster vaccination among those aged 60 and older [29]. Thus, we set the rate of hesitancy toward the booster dose of COVID-19 vaccine as 13% (*p* = 0.13) among old people, calculating the sample size with α as 0.05 and the confidence interval width as 0.1*p* (0.013). In total, 2647 valid questionnaires were expected when using the exact (Clopper–Pearson) method for calculation by the PASS software 15.0 (NCSS LLC., Kaysville, UT, USA). Anonymous questionnaires were randomly allocated among 31 provinces from 25 May to 8 June 2022. The minimum number of valid questionnaires for each province was allocated according to the proportion of the older adults aged 60 or above reported in *the Seventh National Census* [30]. A total of 3321 valid questionnaires were ultimately included in the analysis (Table S1).

### 2.2. Questionnaire Design

This structured questionnaire was divided into 4 sections in total, which consisted of the following parts: (1) sociodemographic characteristics and health status, (2) knowledge of COVID-19 and COVID-19 vaccines, (3) four dimensions of risk perception based on health belief model (HBM), and (4) attitude toward the booster dose of COVID-19. All items in our questionnaire were stated by a panel of experts, including one public health expert and two epidemiologists specializing in infectious diseases. We conducted a pilot study involving 40 old people before it was officially released to test the questionnaire’s validity (Bartlett’s Test of Sphericity: *p* < 0.001). Then the expressions of some items were modified according to the feedback of the respondents, making them easier to understand. The reliability of this questionnaire was confirmed by Cronbach’s alpha coefficient by different dimensions.

The first part comprised questions regarding the sociodemographic characteristics and health status, including region, age, sex, marital status, education level, monthly household income, chronic disease history, and history of COVID-19 vaccination. 

Ten items were set to test participants’ knowledge of COVID-19 and COVID-19 vaccines, ranging from the sources of infection, common symptoms, routes of transmission, high-risk groups, and self-protection measures to questions related to vaccination. Each correct choice got 1 score, and a total of 19 scores and 4 scores were assigned to these two parts, respectively. Then, we divided these scores into three levels, from lowest to highest on average. Cronbach’s alpha coefficient was 0.83.

The health belief model (HBM) is a conceptual framework based on motivation theory, cognitive theory, and expectancy-value theory, which has been widely used to assess public attitudes toward vaccines and predict their behavior toward vaccination [19,20,21]. Four dimensions were extracted in this study to evaluate the risk perception of the elderly population, namely, perceived susceptibility (α = 0.81), perceived severity (α = 0.88), perceived barriers (α = 0.76), and perceived benefit (α = 0.87) [31]. We set 10 questions to investigate the risk perception of older adults, and all questions were answered based on a three-point Likert scale. For questions evaluating perceived susceptibility, scores of 3, 2, and 1 were assigned to “Very Concerned”, “Concerned”, and “Not Concerned”, respectively. Questions involved in the other three dimensions were answered by “Agree (3 points)”, “Not Sure (2 points)”, or “Disagree (1 point)”. Individual’s final level of risk perception was divided equally into three levels (“Low”, “Moderate”, and “High”) according to the total scores in each dimension.

To assess their attitude toward the booster dose of the COVID-19 vaccine, all eligible participants were required to answer the question, “Are you willing to receive the booster dose of COVID-19 vaccine if available?” The hesitancy rate was defined as the proportion of participants who answered “No” or “Not sure”, and if the participants had any concerns about the booster dose, the specific reasons for the reluctance were further asked at the same time.

### 2.3. Data Analysis

Frequencies and percentages were used to describe all categorical variables, consisting of sociodemographic characteristics, health status, knowledge about COVID-19 and the COVID-19 vaccine, and four dimensions of risk perception. An independent Chi-square test was used to compare the vaccine hesitancy rates among different groups stratified by the abovementioned characters. Logistic regression analyses were performed to explore the associations between risk perception and hesitancy rate toward the booster dose among old people (≥60 years old), and we finally constructed four models to examine the robustness of the estimations. Model A is a univariable logistic regression model. Region, age group, sex, marital status, education, and monthly household income were adjusted in model B. All covariates were included along with the other three dimensions of risk perceptions in model C. Additionally, only covariates with significant differences and the other three dimensions of risk perception were contained in model D. Crude odds ratios (cORs) and adjusted odds ratios (aORs) with 95% CIs were calculated to explain the effect size in different risk perception groups.

All statistical analyses were conducted by SPSS 26.0 (IBM SPSS Inc., Armonk, NY, USA) in this study, and a two-sided *p*-value less than 0.05 was regarded as statistically significant.

## 3. Results

### 3.1. Characteristics of the 3321 Participants Aged 60 and Above

A total of 3331 people aged 60 years and older in China were recruited to fill in this questionnaire, of which 3321 participants were eligible for the final analysis (Table 1). Among them, 1097 (33.0%) were aged 70 years old or above, 2290 (69.0%) were married and lived with their spouses, and 1334 (40.1%) at least had an education level of high school. In total, 92.7% of 3321 participants had a history of COVID-19 vaccines, including the single dose (6.9%) and full vaccination (83.4%). Chronic diseases affected the health of almost 2750 (82.8%) older adults. Regarding risk perception, among 3321 participants, 17.8% and 40.3% perceived high susceptibility and severity for the infection of SARS-CoV-2, respectively. About one-third perceived high levels of barriers, while 77.3% of participants have perceived huge benefits of vaccination against COVID-19.

### 3.2. Hesitancy toward the Booster Dose of COVID-19 Vaccine among the Elderly by Characteristics

**Figure 1:**
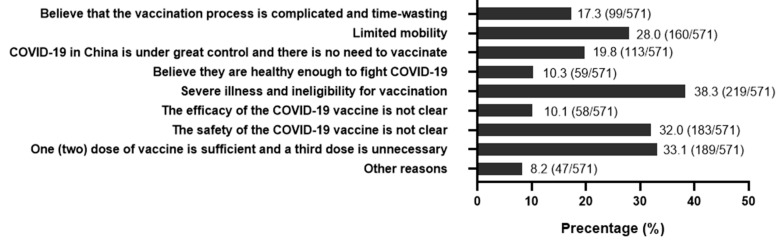
Reasons for responding “No” or “Not sure” regarding willingness to accept the third dose of COVID-19 vaccine (n = 571).

Of 3321 participants, 17.2% (95% CI: 15.9–18.5%) were hesitant about the booster shots of COVID-19 vaccines (Table 1). People who were older, widowed, with lower education level, or without a history of COVID-19 vaccination were prone to have vaccine hesitancy toward the booster dose. In terms of four dimensions of risk perception, vaccine hesitancy differed significantly among different levels of perceived susceptibility, perceived barriers, and perceived benefit (all *p* < 0.05). People with lower perceptions of susceptibility (21.6%) and benefit (45.2%) were less likely to receive a booster dose of vaccination. Perceived barriers had a negative impact on vaccination willingness in older adults. However, no significant difference in vaccination hesitancy was found among groups with different degrees of perceived severity. A total of 571 old people were reluctant about the booster dose of COVID-19 vaccines. Believing that they were ineligible for vaccination due to certain illnesses (38.3%), concern about vaccine safety (32.0%), believing the booster shots were unnecessary (33.1%), and their limitation on movements (28.0%) were the main reasons for vaccine hesitation (Figure 1).

### 3.3. Association between Risk Perception and Hesitancy toward a Booster Dose of COVID-19 Vaccine among Old People

As shown in Table 2, the association between risk perception and hesitancy toward a booster dose of COVID-19 Vaccine among old people was stable in four models. In model A, the vaccine hesitancy toward the booster dose was associated with a low level of perceived susceptibility (cOR = 1.39, 95% CI: 1.06–1.82), higher perceived barriers (moderate: cOR = 3.06, 95% CI: 2.54–3.70; high: cOR = 2.37, 95% CI: 1.44–3.89), and lower level of perceived benefit (low: cOR = 5.40, 95% CI: 2.61–8.08; moderate: cOR = 2.59, 95% CI: 2.11–3.18). Associations above were not substantially altered in model B after controlling for sociodemographic characteristics (region, age, sex, marital status, education, monthly household income per capita). Adjusted by all the selected covariates, people with low perception level of susceptibility (aOR = 1.39, 95% CI: 1.00–1.92) and benefit (low: aOR = 3.31, 95% CI: 2.01–5.45; moderate: aOR = 2.23, 95% CI: 1.75–2.85) were less likely to receiving the booster dose, and the same results were found in the group with a higher level of perceived barriers (moderate: aOR = 2.67, 95% CI: 2.13–3.35; high: aOR = 2.04, 95% CI: 1.14–3.67). Multivariate logistic regression of model D with all statistically significant covariates and three dimensions of risk perception adjusted showed similar association results to the other models.

### 3.4. Subgroup Analyses 

**Figure 2:**
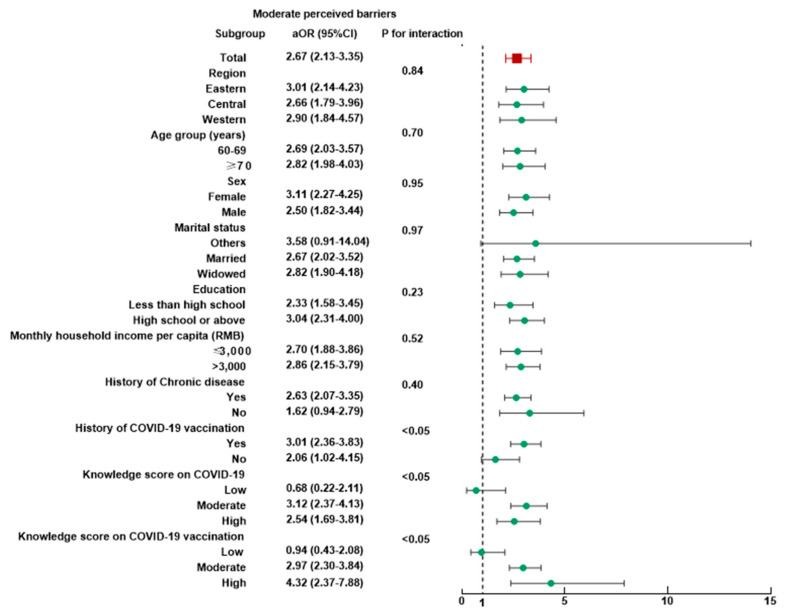
Subgroup analysis of the association between risk perception and hesitancy toward a booster dose of COVID-19 vaccine among 3321 old people with moderate perceived barriers.

Subgroup analyses were performed, and no heterogeneity was found in most subgroups (Table S2). The associations between booster vaccination hesitancy and four dimensions of risk perception were not modified by age group, sex, marital status, education level, or chronic disease history (all *p* for interaction > 0.05). People who perceived low susceptibility in western China (aOR = 2.79, 95% CI: 1.39–5.62) or having lower income (aOR = 2.16, 95% CI: 1.26–3.72) were less likely to accept a booster dose of COVID-19 vaccine (*p* for interaction < 0.05, Figure S1). The association between the perception of moderate barriers and vaccination hesitancy was modified by history of COVID-19 vaccination, knowledge score on COVID-19 and COVID-19 vaccination (all *p* for interaction < 0.05, Figure 2). For people with moderate knowledge scores on COVID-19 vaccination, the highest association (aOR = 2.52, 95% CI: 1.92–3.32) could be found between moderate perceived benefit and the hesitancy rate toward the booster dose of COVID-19 vaccine (*p* for interaction < 0.05, Figure S2).

## 4. Discussion

It is a global consensus that timely vaccination is the most effective, economic, and convenient measure to prevent infectious diseases [5]. To our knowledge, this is the first national study to specifically target people aged 60 or above in China to explore the hesitancy rate toward the booster dose of the COVID-19 vaccine and the association between risk perception and the abovementioned rate. In China, 17.2% (571/3321) were hesitant about the booster shots of COVID-19 vaccines. Concerns about contraindications, vaccine safety, and limited movements were the main reasons for vaccine hesitancy. Nearly a third also thought the booster shots were unnecessary for them. With all covariates adjusted, we found that the vaccine hesitancy was closely related to a lower level of perceived susceptibility and benefit, as well as a higher level of perceived barriers. Results were stable in all four models. Therefore, our findings have important theoretical and practical significance for understanding the vaccination willingness and the subsequent formulation of policies to promote the vaccination coverage of booster dose targeting people aged 60 and older.

Our results showed that 17.2% of older adults were still hesitant about booster vaccination against COVID-19. As of 19 June 2022, 60.7% of the world’s population has been fully vaccinated, while only 26.3 per 100 people have received the booster shots, according to Our World in Data [15]. Meanwhile, for people aged 60 or above in China, nearly 35% still have not administered a booster dose, in which vaccination hesitancy and the time requirement for booster dose might be the main reasons [16,32]. Undoubtedly, promoting the global coverage of initial vaccination and the booster dose of COVID-19 vaccines has become an urgent issue worldwide, which is closely related to the formation of herd immunity in countries and even the world [5,33]. Accounting for 13.5% of the global population, effective immunization in older age groups will play an indispensable role in achieving this goal [34]. To date, there has been no investigation into the hesitancy of booster vaccination and its influencing factors specifically targeting older adults, but our findings can still be compared with specific age groups in some studies [19,35,36]. In a cross-sectional study conducted by Tao-Hsin Tung et al. in Taizhou, China, only 10.3% (85/827) of adults aged 40 and older were unwilling to receive the booster dose [35]. Another large-scale nationwide survey also conducted in China found that 10.56% (30/284) of older adults (≥60 years old) hesitated to receive the booster shots of the COVID-19 vaccine [29]. In Jordan, however, the acceptance rate was only 65.0% among the elderly [36], and 44.6% of Bangladeshis aged over 50 were hesitant to receive booster shots [19]. Thus, our results indicated that the overall acceptance of COVID-19 booster shots among the elderly (≥60 years old) in China is relatively higher than in other countries, but efforts should also be made to remove barriers to vaccination given the large population base.

According to our research, vaccination hesitancy toward the booster dose in the older population was closely associated with low perceived susceptibility, low perceived benefit, and high perceived barriers, but not with perceived severity. As people’s subjective judgment of the characteristics and severity of a particular risk, Risk perception will eventually affect people’s behavior [20,21]. Some studies have demonstrated that fear of infection and trust in the benefits of vaccination could boost the acceptance rates of COVID-19 vaccines [29,37,38,39], which was consistent with our research. A study in Bangladesh showed that the perceived benefit of COVID-19 vaccination (aOR = 0.85, *p* ≤ 0.001) was negatively correlated with hesitation, while perceived barriers (aOR = 1.16, *p* ≤ 0.001) were positively correlated [19]. The association between perceived severity and older adults’ responses to booster shots remains inconsistent [19,29,37,38,39], but evidently, increasing the perceived risk of COVID-19 and the safety and effectiveness of vaccines against COVID-19 under this circumstance is an optimum way to increase the willingness of older adults to receive a booster vaccination. We also found that for older adults, the higher the knowledge scores on COVID-19 and COVID-19 vaccines, the lower the vaccination hesitancy toward the booster shots. A survey conducted in southern Italy suggested that what people over 65 knew about COVID-19 could change their behavior during this pandemic [24]. Chen et al. indicated that the practice of prevention behavior among the elderly in China was positively correlated with the scores of knowledge related to COVID-19 [23]. At different stages of this pandemic, knowledge about COVID-19 and vaccines have contributed to the vaccination willingness of older populations in China, Brazil, Malaysia, Singapore, Colombia, and other countries [40,41,42,43,44]. Therefore, it is of practical significance to improve the understanding of the elderly on their own susceptibility, effectiveness, and safety of vaccines through various publicity and education ways (such as social media, offline lectures, etc.) to promote valid herd immunity.

Unlike young people, older people are more worried and concerned about booster shots. In this survey, 38.3% of the elderly worried that they were not eligible for booster shots for their existing diseases. Evidently, advanced age and comorbidities such as hypertension, diabetes, cardiovascular disease, and chronic respiratory disease were risk factors for SARS-CoV-2 infection and poor prognosis [6,7]. People with chronic diseases that are well controlled by drugs are usually not considered contraindicated groups for COVID-19 vaccines [45]. People with other diseases or special circumstances should consult professional health workers in detail to determine whether they are eligible for vaccination [45]. In addition, in order to provide longer-lasting immunity and greater protection against the evolving SARS-CoV-2 variants, booster vaccination is a natural choice to build and consolidate herd immunity, especially in high-risk groups such as older adults. Whether from the perspective of individuals or herds, the benefits of vaccinating old people based on their own conditions far outweigh the risks, including the booster dose. Therefore, we should realistically reduce or try to solve the causes of vaccination hesitancy among the elderly in order to improve vaccination coverage among old people. Measures such as simplifying the vaccination process, providing door-to-door vaccination services, and arranging medical professionals to answer elderly people’s doubts about whether they are eligible for vaccination are also supposed to be promoted.

As with other online cross-sectional surveys, our study also has some limitations. First, selection bias may exist. Considering the accessibility of online surveys for the elderly, questionnaires were answered only by Internet users. Moreover, some elderly people who had difficulty answering questions using electronic devices may seek help from young people around them and answer questions orally through young people’s dictation. This may also increase the selection bias of this study. Although we have set up tips at the end of each question and repeatedly emphasized that the subjective questions were supposed to be answered by the elderly orally, we cannot completely rule out proxy answers. However, under the current situation, it is urgent to understand the vaccination willingness toward the booster shots and promote vaccination coverage among the elderly. In addition, people’s acceptance of the booster dose of the COVID-19 vaccine was measured only by self-report, and we were unable to develop a standard scale to assess their willingness. Third, this survey was only conducted in China and based on a specific theoretical model, so results need to be interpreted carefully when extrapolated to other countries or compared with other models. Since this was an online survey, our depth was limited to some extent. Our results were only a crude supplement to the current research gap, and we hope that large-scale offline surveys with more participants will be implemented as soon as feasible. Moreover, this is only a cross-sectional study, which cannot fully verify the relationship between risk perception and vaccination hesitancy from a causal perspective.

## 5. Conclusions

Of 3321 participants, 17.2% were hesitant about the booster dose of the COVID-19 vaccine, and it was closely associated with a lower level of perceived susceptibility and benefit, as well as a higher level of perceived barriers. Concerns about contraindications, vaccine safety, and limited movements were the main reasons for vaccine hesitancy. Under this circumstance, it is urgent to improve the understanding of the elderly on their own susceptibility and vulnerability, reasonably judge whether they could be vaccinated, and clear the obstacles to vaccination. Therefore, our results have important theoretical and practical implications for the subsequent promotion of the coverage of booster doses in the elderly population.

## Figures and Tables

**Table 1:** Hesitancy toward the booster dose of COVID-19 vaccine among 3321 old people in China by characteristics.

Characteristics	N (%)	Hesitancy toward the Booster Dose of COVID-19 Vaccine to Children		*p*-Value
		N (%)	95%CI	
**Total**	3321 (100)	571 (17.2)	15.9–18.5	
**Sociodemographic Characteristics**				
**Region**				0.96
Eastern	1443 (43.5)	248 (17.2)	15.3–19.2	
Central	1016 (30.6)	177 (17.4)	15.2–19.8	
Western	862 (26.0)	146 (16.9)	14.5–19.6	
**Age Group (Years)**				<0.05 *
60–64	982 (29.6)	132 (13.4)	11.4–15.7	
65–69	1242 (37.4)	185 (14.9)	13.0–17.0	
≥70	1097 (33.0)	254 (23.2)	20.7–25.7	
**Sex**				0.76
Female	1731 (52.1)	301 (17.4)	15.7–19.2	
Male	1590 (47.9)	270 (17.0)	15.2–18.9	
**Marital Status ^a^**				<0.05 *
Married	2290 (69.0)	349 (15.2)	13.8–16.8	
Widowed	926 (27.9)	203 (21.9)	19.3–24.7	
Others	105 (3.2)	19 (18.1)	11.6–26.3	
**Education ^b^**				<0.05 *
Beyond high school	499 (15.0)	71 (14.2)	11.4–17.5	
High school	835 (25.1)	120 (14.4)	12.1–16.9	
Junior high school	905 (27.3)	154 (17.0)	14.7–19.6	
Primary and below	1082 (32.6)	226 (20.9)	18.5–23.4	
**Monthly Household Income Per Capita (RMB)**				0.30
≤1500	503 (15.1)	101 (20.1)	16.8–23.7	
1501–3000	719 (21.7)	123 (17.1)	14.5–20.0	
3001–5000	1024 (30.8)	179 (17.5)	15.2–19.9	
5001–10,000	791 (23.8)	124 (15.7)	13.3–18.3	
>10,000	284 (8.6)	44 (15.5)	11.6–20.0	
**Health Status**				
**History of Chronic Disease**				0.22
No	571 (17.2)	88 (15.4)	12.6–18.5	
Yes	2750 (82.8)	483 (17.6)	16.2–19.0	
**History of COVID-19 Vaccination ^c^**				<0.05 *
No vaccination	322 (9.7)	212 (65.8)	60.5–70.9	
Single dose	230 (6.9)	84 (36.5)	30.5–42.9	
Full vaccination	2769 (83.4)	275 (9.9)	8.9–11.1	
**Knowledge Factors**				
**Knowledge Score on COVID-19**				<0.05 *
Low (score 0–6)	120 (3.6)	39 (32.5)	24.6–41.2	
Moderate (score 7–13)	1762 (53.1)	358 (20.3)	18.5–22.2	
High (score 14–19)	1439 (43.3)	174 (12.1)	10.5–13.9	
**Knowledge Score on COVID-19 Vaccination**				<0.05 *
Low (score 0–1)	247 (7.4)	59 (23.9)	18.9–29.5	
Moderate (score 2–3)	2461 (74.1)	421 (17.1)	15.7–18.6	
High (score 4)	613 (18.5)	91 (14.8)	12.2–17.8	
**Risk Perception**				
**Perceived Susceptibility**				<0.05 *
Low (score 2–3)	814 (24.5)	176 (21.6)	18.9–24.5	
Moderate (score 4–5)	1916 (57.7)	297 (15.5)	13.9–17.2	
High (score 6)	591 (17.8)	98 (16.6)	13.8–19.7	
**Perceived Severity**				0.55
Low (score 2–3)	293 (8.8)	49 (16.7)	12.8–21.3	
Moderate (score 4–5)	1688 (50.8)	280 (16.6)	14.9–18.4	
High (score 6)	1340 (40.3)	242 (18.1)	16.1–20.2	
**Perceived Barriers**				<0.05 *
Low (score 3–4)	2275 (68.5)	270 (11.9)	10.6–13.2	
Moderate (score 5–7)	955 (28.8)	279 (29.2)	26.4–32.2	
High (score 8–9)	91 (2.7)	22 (24.2)	16.3–33.7	
**Perceived Benefit**				<0.05 *
Low (score 3–5)	104 (3.1)	47 (45.2)	35.9–54.8	
Moderate (score 6–7)	649 (19.5)	184 (28.4)	25.0–31.9	
High (score 8–9)	2568 (77.3)	340 (13.2)	12.0–14.6	

* *p* < 0.05. ^a^ “Married” referred to the married old people whose spouses were still alive. ^b^ “High school” included high school education and technical secondary school education. ^c^ “No vaccination” referred to people who were not vaccinated at all; “Single dose” meant only received one dose of inactivated vaccine. “Full vaccination” referred to complete vaccination without a booster dose.

**Table 2:** The association between risk perception and the hesitancy toward the booster dose of COVID-19 vaccine to children among 3321 old people in China.

	Model A		Model B		Model C		Model D	
	Crude Odds Ratio (95% CI)	*p*-Value	Adjusted Odds Ratio (95% CI)	*p*-Value	Adjusted Odds Ratio (95% CI)	*p*-Value	Adjusted Odds Ratio (95% CI)	*p*-Value
**Perceived Susceptibility**								
Low (score 2–3)	1.39 (1.06–1.82)	<0.05 *	1.34 (1.01–1.77)	<0.05 *	1.39 (1.00–1.92)	<0.05 *	1.39 (1.00–1.93)	<0.05 *
Moderate (score 4–5)	0.92 (0.72–1.18)	0.53	0.89 (0.69–1.15)	0.37	1.08 (0.81–1.45)	0.59	1.09 (0.81–1.46)	0.56
High (score 6)	Reference		Reference		Reference		Reference	
**Perceived Severity**								
Low (score 2–3)	0.91 (0.65–1.28)	0.59	0.94 (0.67–1.32)	0.72	0.97 (0.65–1.44)	0.88	0.97 (0.66–1.44)	0.90
Moderate (score 4–5)	0.90 (0.75–1.09)	0.29	0.92 (0.76–1.12)	0.40	0.92 (0.74–1.15)	0.48	0.93 (0.74–1.16)	0.50
High (score 6)	Reference		Reference		Reference		Reference	
**Perceived Barriers**								
Low (score 3–4)	Reference		Reference		Reference		Reference	
Moderate (score 5–7)	3.06 (2.54–3.70)	<0.05 *	3.01 (2.53–3.72)	<0.05 *	2.67 (2.13–3.35)	<0.05 *	2.66 (2.13–3.33)	<0.05 *
High (score 8–9)	2.37 (1.44–3.89)	<0.05 *	2.50 (1.51–4.14)	<0.05 *	2.04 (1.14–3.67)	<0.05 *	2.02 (1.13–3.62)	<0.05 *
**Perceived Benefit**								
Low (score 3–5)	5.40 (2.61–8.08)	<0.05 *	5.52 (3.65–8.32)	<0.05 *	3.31 (2.01–5.45)	<0.05 *	3.21 (1.95–5.26)	<0.05 *
Moderate (score 6–7)	2.59 (2.11–3.18)	<0.05 *	2.57 (2.09–3.17)	<0.05 *	2.23 (1.75–2.85)	<0.05 *	2.21 (1.73–2.82)	<0.05 *
High (score 8–9)	Reference		Reference		Reference		Reference	

* *p* < 0.05, Model A is a univariate logistic regression model, using crude odds ratios (cORs) to explain the vaccine hesitancy in different risk perception groups. Region, age group, sex, marital status, education, and monthly household income per capita were adjusted in model B. In model C, we adjusted the rest covariates based on model B—history of chronic disease, history of COVID-19 vaccination, knowledge score on COVID-19, knowledge score on COVID-19 vaccination, as well as the other three aspects of risk perceptions. Model D only contained the significant covariates in Pearson χ2 test and the other three risk perceptions.

## Data Availability

All data in the study are available from the corresponding author by request.

## Supplementary Materials

The following supporting information can be downloaded at: https://www.mdpi.com/article/10.3390/vaccines10071112/s1, Figure S1: Subgroup analysis of the association between risk perception and hesitancy toward a booster dose of COVID-19 vaccine among 3321 old people with low perceived susceptibility.; Figure S2: Subgroup analysis of the association between risk perception and hesitancy toward a booster dose of COVID-19 vaccine among 3321 old people with moderate perceived benefit; Table S1: Collection of valid questionnaires by region in mainland China; Table S2: Subgroup analysis of the association between risk perception and hesitancy toward the booster dose of COVID-19 vaccine among old people.
