# Supplementary material for: The Association between Risk Perception and Hesitancy toward the Booster Dose of COVID-19 Vaccine among People Aged 60 Years and Older in China
Source: Vaccines (Basel). 2022 Jul 12;10(7):1112. doi: 10.3390/vaccines10071112 (PMC9317690; doi:10.3390/vaccines10071112)
Supplement: Supplementary file 1 [file vaccines-10-01112-s001.zip › vaccines-1810554-Supplementary file.pdf]

Supplementary File

Table S1. Collection of valid questionnaires by region in mainland China.

Regions	Number	Proportion
Guangdong Province	190	5.72%
Shandong Province	260	7.83%
Henan Province	223	6.71%
Jiangsu Province	230	6.93%
Sichuan Province	224	6.74%
Hebei Province	190	5.72%
Hunan Province	161	4.85%
Zhejiang Province	149	4.49%
Anhui Province	140	4.22%
Hubei Province	145	4.37%
Guangxi Province	107	3.22%
Jiangxi Province	95	2.86%
Liaoning Province	140	4.22%
Fujian Province	85	2.56%
Shaanxi Province	96	2.89%
Guizhou Province	76	2.29%
Shanxi Province	90	2.71%
Chongqing	89	2.68%
Heilongjiang Province	92	2.77%
Yunnan Province	90	2.71%
the Nei Monggol Autonomous Region	61	1.84%
Jilin Province	70	2.11%
Shanghai	80	2.41%
Gansu Province	54	1.63%
Beijing	60	1.81%
the Xinjiang Uygur Autonomous Region	36	1.08%
Tianjin	39	1.17%
Hainan Province	20	0.60%
Ningxia Province	15	0.45%
Tibet	4	0.12%
Qinghai Province	10	0.30%

Table S2. Subgroup analysis of the association between risk perception and hesitancy toward the booster dose of COVID-19 vaccine among old people.

**Subgroup**	**Low Perceived Susceptibility**	***P* for Interaction**	**Moderate Perceived Susceptibility**	***P* for Interaction**	**Low Perceived Severity**	***P* for Interaction**	**Moderate Perceived Severity**	***P* for Interaction**	**Moderate Perceived Barriers**	***P* for Interaction**	**High Perceived Barriers**	***P* for Interaction**	**Low Perceived Benefit**	***P* for Interaction**	**Moderate Perceived Benefit**	***P* for Interaction**
**Total**	1.39 (1.00–1.92)		1.08 (0.81–1.45)		0.97 (0.65–1.44)		0.92 (0.74–1.15)		2.67 (2.13–3.35)		2.04 (1.14–3.67)		3.31 (2.01–5.45)		2.23 (1.75–2.85)	
**Sociodemographic characteristics**																
**Region**		**<0.05***		0.31		0.44		0.88		0.84		0.60		0.41		0.70
Eastern	0.84 (0.52–1.36)		0.86 (0.58–1.29		0.89 (0.49–1.65)		0.99 (0.71–1.38)		3.01 (2.14–4.23)		3.01 (1.42–6.36)		2.14 (0.99–4.64)		2.38 (1.67–3.38)	
Central	1.64 (0.90–3.00)		1.31 (0.75–2.29)		1.37 (0.71–2.66)		0.85 (0.56–1.27)		2.66 (1.79–3.96)		2.01 (0.63–6.47)		4.34 (1.74–10.83)		2.12 (1.37–3.28)	
Western	2.79 (1.39–5.62)		1.35 (0.71–2.57)		1.28 (0.60–2.70)		0.97 (0.62–1.51)		2.90 (1.84–4.57)		1.42 (0.34–5.92)		4.48 (1.82–11.07)		1.93 (1.16–3.22)	
**Age group (years)**		0.07		0.08		0.46		0.72		0.70		0.49		0.64		0.58
60–69	1.11 (0.74–1.66)		0.91 (0.64–1.30)		1.25 (0.78–2.00)		0.98 (0.74–1.31)		2.69 (2.03–3.57)		2.67 (1.36–5.23)		3.64 (2.00–6.63)		2.14 (1.58–2.91)	
≥70	2.16 (1.24–3.75)		1.55 (0.94–2.56)		0.89 (0.46–1.73)		0.88 (0.62–1.25)		2.82 (1.98–4.03)		1.31 (0.43–4.02)		2.55 (1.11–5.82)		2.36 (1.60–3.47)	
**Sex**		0.24		0.14		0.96		0.48		0.95		0.06		0.18		0.81
Female	1.88 (1.17–3.01)		1.44 (0.95–2.18)		1.15 (0.67–2.00)		1.03 (0.76–1.41)		3.11 (2.27–4.25)		4.49 (2.07–9.74)		2.37 (1.18–4.76)		2.27 (1.62–3.17)	
Male	1.06 (0.68–1.67)		0.82 (0.54–1.24)		0.95 (0.56–1.63)		0.82 (0.59–1.13)		2.50 (1.82–3.44)		1.05 (0.41–2.67)		4.46 (2.21–9.02)		2.25 (1.59–3.18)	
**Marital status**		0.71		0.99		0.20		0.45		0.97		0.33		0.91		0.47
Others	0.70 (0.11–4.41)		0.81 (0.13–4.85)		1.45 (0.17–12.36)		0.51 (0.11–2.42)		3.58 (0.91–14.04)		–		–		2.41 (0.57–10.26)	
Married	1.34 (0.89–2.00)		1.05 (0.73–1.51)		1.36 (0.86–2.13)		1.04 (0.78–1.37)		2.67 (2.02–3.52)		1.92 (0.96–3.82)		3.04 (1.71–5.40)		2.49 (1.85–3.35)	
Widowed	1.46 (0.80–2.65)		1.04 (0.62–1.74)		0.59 (0.26–1.32)		0.77 (0.53–1.13)		2.82 (1.90–4.18)		2.49 (0.81–7.68)		3.39 (1.29–8,92)		1.84 (1.19–2.84)	
**Education**		0.12		0.29		0.44		0.33		0.23		0.33		0.95		0.46
Less than high school	1.02 (0.60–1.73)		0.89 (0.56–1.42)		1.41 (0.74–2.67)		1.10 (0.76–1.61)		2.33 (1.58–3.45)		1.65 (0.67–4.05)		3.81 (1.65–8.77)		2.74 (1.81–4.13)	
High school or above	1.64 (1.09–2.48)		1.21 (0.84–1.75)		0.95 (0.58–1.53)		0.86 (0.66–1.14)		3.04 (2.31–4.00)		2.91 (1.38–6.15)		3.06 (1.67–5.63)		2.04 (1.52–2.74)	

**Monthly household income per capita (RMB)**		**<0.05***		0.09		0.08		0.64		0.52		0.58		0.24		0.40
≤3,000	2.16 (1.26–3.72)		1.51 (0.91–2.49)		1.56 (0.87–2.77)		1.00 (0.69–1.44)		2.70 (1.88–3.86)		2.18 (0.85–5.58)		2.43 (1.14–5.18)		2.57 (1.76–3.77)	
>3,000	1.07 (0.71–1.61)		0.89 (0.62–1.27)		0.82 (0.49–1.38)		0.90 (0.68–1.19)		2.86 (2.15–3.79)		2.38 (1.16–4.90)		4.53 (2.33–8.80)		2.02 (1.48–2.76)	
**Health status**																
**History of chronic disease**		1.00		0.75		0.51		0.63		0.40		0.91		0.58		0.45
Yes	1.38 (0.97–1.95)		1.09 (0.80–1.49)		1.14 (0.76–1.73)		0.97 (0.76–1.23)		2.63 (2.07–3.35)		2.14 (1.15–3.98)		3.00 (1.76–5.12)		2.31 (1.78–3.00)	
No	1.25 (0.53–2.95)		0.81 (0.36–1.82)		0.92 (0.33–2.53)		0.78 (0.43–1.42)		3.29 (1.83–5.92)		2.76 (0.61–12.35)		4.37 (1.25–15.27)		1.70 (0.89–3.24)	
**History of COVID–19 vaccination**		0.64		0.08		0.27		0.70		**<0.05***		0.72		0.97		0.14
Yes	1.27 (0.89–1.81)		0.94 (0.68–1.29)		1.20 (0.80–1.80)		0.96 (0.75–1.24)		3.01 (2.36–3.83)		2.29 (1.25–4.21)		3.19 (1.88–5.41)		2.29 (1.77–2.97)	
No	2.06 (1.02–4.15)		1.77 (0.93–3.35)		0.64 (0.21–1.90)		1.00 (0.59–1.67)		1.62 (0.94–2.79)		1.31 (0.23–7.39)		3.75 (1.01–13.98)		1.57 (0.85–2.90)	
**Knowledge score on COVID–19**		0.45		0.15		0.78		0.25		**<0.05***		0.35		0.15		0.32
Low	4.00 (0.54–29.2)		6.99 (0.94–51.99)		6.32 (1.08–36.85)		3.16 (0.81–12.43)		0.68 (0.22–2.11)		0.82 (0.05–14.50)		2.79 (0.45–17.08)		2.00 (0.61–6.62)	
Moderate	1.21 (0.81–1.82)		0.91 (0.63–1.31)		0.92 (0.56–1.53)		0.94 (0.71–1.24)		3.12 (2.37–4.13)		2.69 (1.32–5.47)		2.40 (1.34–4.31)		2.37 (1.76–3.18)	
High	1.92 (1.07–3.45)		1.37 (0.82–2.30)		1.14 (0.57–2.31)		0.90 (0.60–134)		2.54 (1.69–3.81)		1.68 (0.54–5.19)		6.60 (2.33–18.70)		1.67 (1.04–2.68)	
**Knowledge score on COVID–19 vaccination**		0.80		0.76		0.80		0.18		**<0.05***		0.66		0.17		**<0.05***
Low	1.06 (0.34–3.25)		1.18 (0.42–3.31)		1.08 (0.37–3.10)		0.79 (0.36–1.74)		0.94 (0.43–2.08)		2.25 (0.38–13.54)		1.44 (0.45–4.61)		1.33 (0.57–3.08)	
Moderate	1.36 (0.93–1.98)		1.01 (0.72–1.42)		1.15 (0.73–1.80)		0.87 (0.68–1.13)		2.97 (2.30–3.84)		2.54 (1.27–5.08)		4.09 (2.20–7.60)		2.52 (1.92–3.32)	
High	2.62 (1.11–6.16)		1.68 (0.80–3.52)		1.10 (0.35–3.47)		1.51 (0.86–2.66)		4.32 (2.37–7.88)		1.54 (0.35–6.75)		2.20 (0.63–7.64)		1.95 (0.99–3.81)	

* p <0.05, effect size was aOR and 95%CI

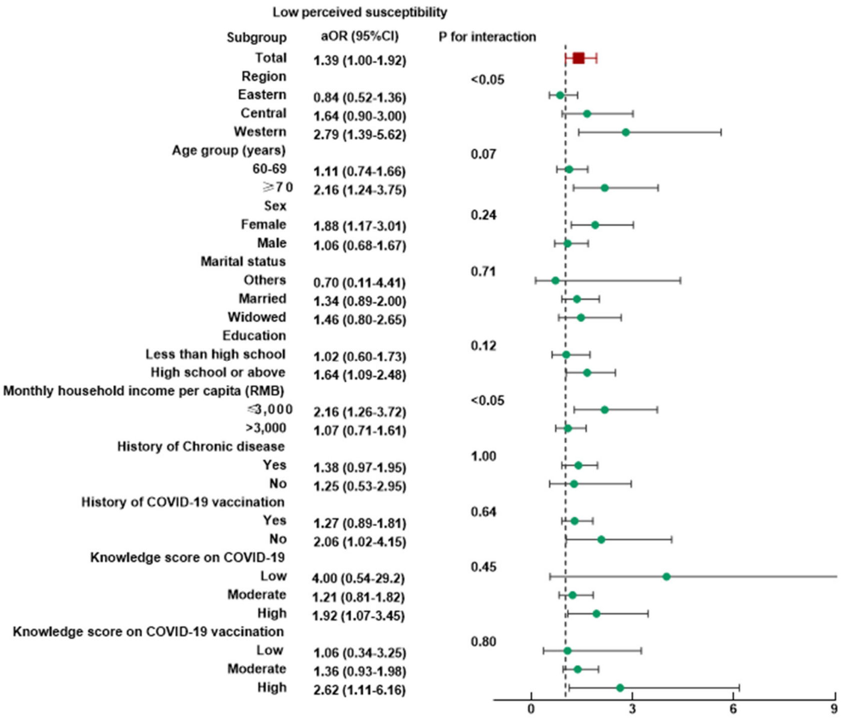

Figure S1. Subgroup analysis of the association between risk perception and hesitancy toward a booster dose of COVID-19 vaccine among 3,321 old people with low perceived susceptibility.

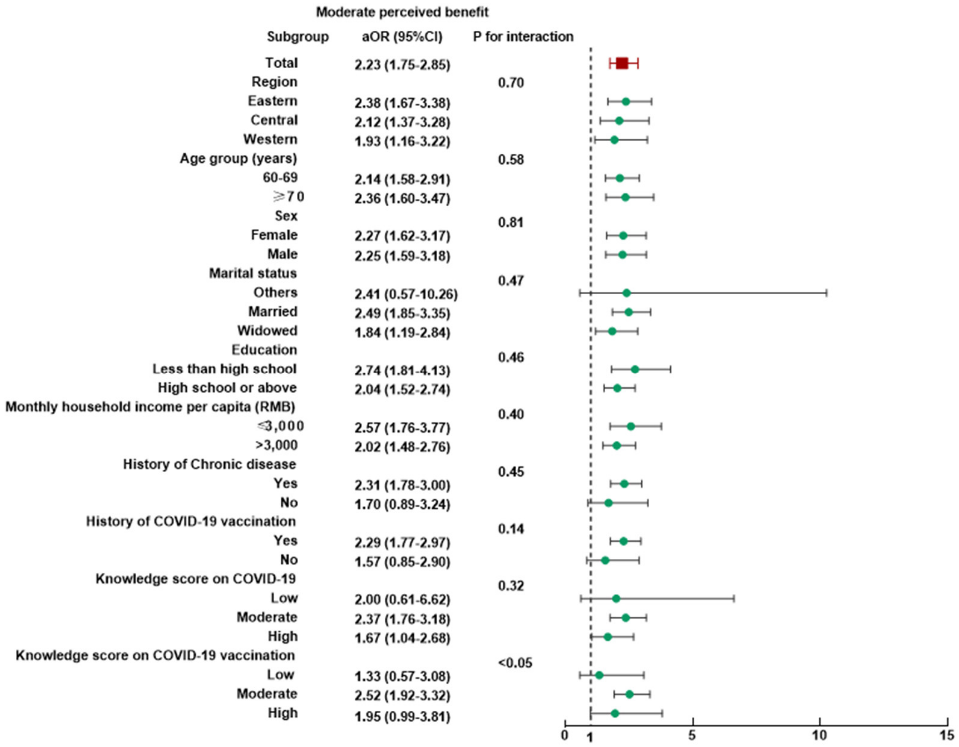

Figure S2. Subgroup analysis of the association between risk perception and hesitancy toward a booster dose of COVID-19 vaccine among 3,321 old people with moderate perceived benefit.
